# Assessment of the clinical and analytical performance of the Aptima SARS-CoV-2 assay using the VALCOR protocol

**DOI:** 10.1186/s12985-023-01986-4

**Published:** 2023-02-24

**Authors:** Sharonjit K. Dhillon, Cindy Simoens, Lize Cuypers, Jannes Bode, Jesper Bonde, Philippe Corbisier, Clementina E. Cocuzza, Marc Van Ranst, Marc Arbyn

**Affiliations:** 1 Unit of Cancer Epidemiology, Belgian Cancer Centre, Sciensano, J. Wytsmanstreet 14, B1050 Brussels, Belgium; 2 National Reference Centre for Respiratory Pathogens, Department of Laboratory Medicine, University Hospitals Leuven, Leuven, Belgium; 3 Molecular Pathology Laboratory, Department of Pathology, Copenhagen University Hospital -Amager and Hvidovre Hospital, Copenhagen, Denmark; 4 European Commission, Joint Research Centre, Directorate F – Health, Consumers and Reference Materials, Geel, Belgium; 5 Laboratory of Clinical Microbiology and Virology, Department of Medicine and Surgery, University of Milano – Bicocca, Monza, Italy; 6 Laboratory of Clinical and Epidemiological Virology, Department of Microbiology, Immunology and Transplantation, Rega Institute for Medical Research, KU Leuven, Leuven, Belgium; 7 Department of Human Structure and Repair, Faculty of Medicine and Health Sciences, University of Ghent, Ghent, Belgium

**Keywords:** SARS-CoV-2, Diagnostic test accuracy, RT-PCR, Transcription mediated amplification, COVID-19, Test validation, Quality control, Aptima, VALCOR

## Abstract

**Background:**

The COVID-19 pandemic highlighted the importance of diagnostic testing against curbing the spread of SARS-CoV-2. The urgent need and scale for diagnostic tools resulted in manufacturers of SARS-CoV-2 assays receiving emergency authorization that lacked robust analytical or clinical evaluation. As it is highly likely that testing for SARS-CoV-2 will continue to play a central role in public health, the performance characteristics of assays should be evaluated to ensure reliable diagnostic outcomes are achieved.

**Methods:**

VALCOR or “VALidation of SARS-CORona Virus-2 assays” is a study protocol designed to set up a framework for test validation of SARS-CoV-2 virus assays. Using clinical samples collated from VALCOR, the performance of Aptima SARS-CoV-2 assay was assessed against a standard comparator assay. Diagnostic test parameters such as sensitivity, specificity and overall per cent agreement were calculated for the clinical performance of Aptima SARS-CoV-2 assay.

**Results:**

A total of 180 clinical samples were tested with an addition of 40 diluted clinical specimens to determine the limit of detection. When compared to the standard comparator assay Aptima had a sensitivity of 100.0% [95% CI 95.9–100.0] and specificity of 96.7% [95% CI 90.8–99.3]. The overall percent agreement was 98.3% with an excellent Cohen’s coefficient of κ = 0.967 [95% CI 0.929–1.000]. For the limit of detection, Aptima was able to detect all of the diluted clinical samples.

**Conclusion:**

In conclusion. validation of Aptima SARS-CoV-2 assay using clinical samples collated through the VALCOR protocol showed excellent test performance. Additionally, Aptima demonstrated high analytical sensitivity by detecting all diluted clinical samples corresponding to a low limit of detection.

## Background

The severe acute respiratory syndrome coronavirus 2 (SARS-CoV-2) is a highly transmissible coronavirus first detected in Wuhan, China [1, 2]. Three months later, the World Health Organization (WHO) declared the outbreak a global pandemic in March 2020. Since its emergence, the SARS-CoV-2 has infected millions of individuals and caused over 6.3 million deaths worldwide [3]. Individuals confirmed with coronavirus disease (COVID-19) show a range of symptoms from mild to severe respiratory failure. Whilst the virus is known to be associated with high mortality, specifically in vulnerable populations, many individuals may be asymptomatic but still have the ability to transmit the virus to others [4, 5].

The COVID-19 pandemic instigated an exceptional need to increase large-scale testing globally. Despite steady efforts in vaccination against SARS-CoV-2, waning immunity, the emergence of vaccine-evasive variants and limited access to vaccines in lower and middle-income countries as well as the continued high viral circulation in populations are factors that continue to pose a threat for recurrent outbreaks. Hence, clinical testing remains one of the key approaches in the COVID-19 response to control the spread and infection rate of the virus [6].

Curbing large-scale outbreaks requires the availability of validated high-throughput diagnostic assays that are reliable and accurate. Assays that detect the presence of the virus itself include nucleic acid amplification tests (NAATs). NAATs detect targeted sequences of the SARS-CoV-2 genome from respiratory samples through different amplification methods. The most commonly used and often considered the gold standard method of such NAATs is the reverse transcriptase polymerase chain reaction (RT-PCR). Despite having an advantage in terms of analytical sensitivity and accuracy, high-throughput assays using RT-PCR need to be performed in well-equipped settings, require skilled personnel and have long turnover times [6–8]. These factors become a major challenge for laboratories to provide timely results for quick identification and isolation of infected individuals which is key to curbing any infectious disease. Assays that utilize other amplification methods such as transcription-mediated amplification (TMA) provide faster results for timely diagnosis. The difference between TMA and RT-PCR is that the isothermal amplification of targeted RNA sequences in TMA is achieved using a constant temperature whereas the RT-PCR method requires a thermal cycler which changes the reaction temperatures repeatedly. Transcription-mediated amplification (TMA) involves the isothermal amplification of rRNA by reverse transcription and subsequent generation of numerous transcripts by RNA polymerase. Following amplification, these RNA copies are hybridized with a complementary oligonucleotide probe for detection via a chemiluminescent tag or a fluorescent-labelled molecular beacon [9].

In May 2020, Hologic^®^ Aptima SARS-CoV-2 assay (hereinafter referred to as 'Aptima') based on TMA received Emergency Use Authorization (EUA-200734) from the Food and Drug Administration (FDA) for the qualitative detection of SARS-CoV-2 ribonucleic acid (RNA) from respiratory samples. The Aptima SARS-CoV-2 assay (Hologic^®^ Panther System) is run on the Panther platform, an automated platform that has a fast turnaround time and can test up to 1200 samples per day. The one-step isothermal condition allows a faster turnaround time than RT-PCR. The main advantage of the Aptima is that the TMA can be performed in the lysis buffer without prior purification of the RNA producing faster results with a low sensitivity enabling pooling testing [10].

Due to the nature of the pandemic, many assays received an EUA to expedite the otherwise stringent criteria diagnostics assays have to fulfil in order to receive full approval. EUA was essential to address the need to make diagnostic assays available quickly however it is still vital that such commercial assays become FDA cleared or approved according to a systematic standardized validation framework. In this present study, we aim to evaluate the test performance of the automated Aptima SARS-CoV-2 assay (Hologic^®^ Panther System) using clinical samples collated according to the VALCOR protocol [11] (acronym for “VALidation of SARS-CORona Virus-2 assays”).

## Materials and methods

### Composition of the VALCOR panel

VALCOR is a protocol designed to set up a framework for test validation of SARS-CoV-2 virus assays and is inspired by the principles of VALGENT (VALidation of HPV GENotyping Test), which is a world-widely recognized forum for HPV test comparison and validation [12]. The full protocol of VALCOR is described elsewhere [11].

**Fig. 1:**
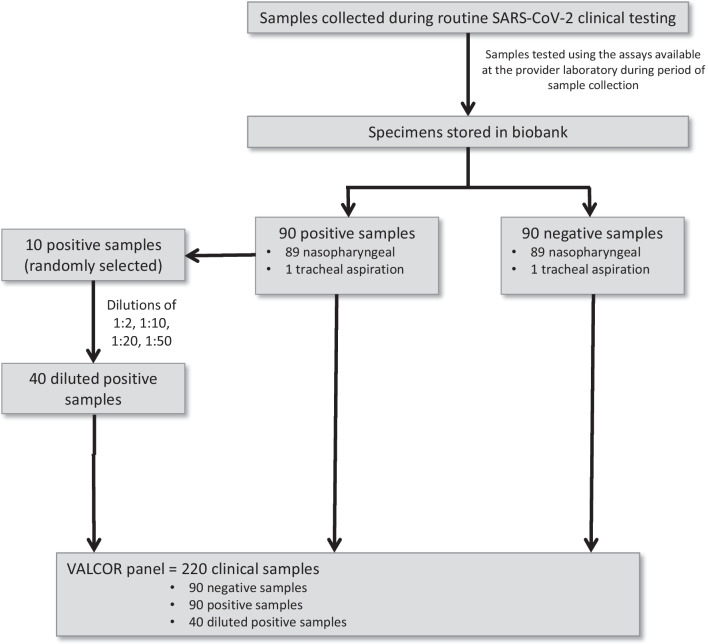
Overview of samples that were included in the VALCOR panel

The VALCOR panel was collated using stored biobank samples collected as part of routine clinical testing from the National Reference Laboratory For Respiratory Pathogens, Department of Laboratory Medicine, University Hospitals Leuven, Belgium where they had been stored at − 20 °C. The panel consists of a total of 220 clinical specimens (180 non-diluted and 40 diluted samples) collected between March 2020 and February 2021. The panel included one sample per patient from 180 patients. Among them, 90 samples were positive for SARS-CoV-2 and 90 samples were negative for SARS-CoV-2. 178 were nasopharyngeal samples and two samples were tracheal aspirations. Samples were suspended in Universal Transport Medium (UTM, N = 98), Viral Transport Medium (VTM, N = 51) and Phosphate Buffered Saline (PBS N = 31). The limit of detection (LoD) of the Aptima assay was determined by testing serial dilutions of ten samples (randomly selected) from the 90 positive samples. The ten samples were diluted at 1:2, 1:10, 1:20 and 1:50 in UTM. The overview of the VALCOR panel is presented in Fig. 1.

This study was approved by the Ethics Committee Research (EC Research) of University Hospitals Leuven (UZ Leuven) and KU Leuven under the registration reference number S64233.

### Testing of samples with index assay

Testing with the Aptima SARS-CoV-2 assay VALCOR panel was carried out at Clinical Laboratory Department, Hospital Nostra Senyora de Meritxell, Escaldes-Engordany, Andorra on July 7, 2021. Specimens were transported on dry ice with constant temperature monitoring prior to being subject to nucleic acid extraction and subsequent testing.

Aptima combines the technologies of target capture and transcription-mediated amplification. The assay is used on the automated, random access Panther system (Hologic) and can provide results within 3.5 h and process 120 samples per run with continuous loading. The assay was performed according to manufacturer instructions: 0.5 mL of sample was pipetted into a Hologic lysis tube and loaded directly on the instrument. Aptima utilizes magnetic bead-based target capture, isothermal TMA of RNA, and dual kinetic acridinium ester-labelled probe hybridization for the isolation, amplification, and detection of an internal process control RNA and two unique sequences within the ORF1ab region of the SARS-CoV-2 viral genome. The outcomes of testing are recorded as “positive”, “negative” or “invalid”.

### Testing of samples with a standard comparator assay

Initial testing of samples was performed at the National Reference Laboratory For Respiratory Pathogens, Department of Laboratory Medicine, University Hospitals Leuven between 1st April 2020 and 15th January 2021. Classification of positive and negative samples was provided by initial test results using different assays that were used in the period of patient enrollment. As the samples were analyzed in the context of routine diagnostics during a time when there were shortages of assays and reagents as well as great demand for high testing capacity, samples were tested using different platforms and assays depending on the availability of the assays in the laboratory at that time. In order to have a common reference assay, all specimens were retested at the national reference laboratory using TaqPath™ COVID-19 CE-IVD RT-PCR Kit (ThermoFisher Scientific) (hereinafter referred to as 'TaqPath') as the chosen reference assay, after the composition of the VALCOR panel. Discordant samples (initial testing vs TaqPath) were retested on Alinity m SARS-CoV-2 assay (Abbott Laboratories, Chicago, IL, USA) and/or the GeneXpert (Cepheid, Sunnyvale, California, USA) for additional verification.

TaqPath is an in vitro nucleic acid amplification test for the detection of SARS-CoV-2 and is one of the main assays used for testing of SARS-CoV-2 in Belgium. At UZ Leuven, the analysis is performed using a Quantstudio 7 Flex thermocycler, preceded by extraction using a KingFisher Flex in combination with the MagMax Viral Pathogen II extraction kit (ThermoFisher Scientific). The analysis is embedded within the KingFisher High Throughput platform, which consists of two pipetting robots (TECAN EVO), two KingFisher extraction machines and two QuantStudio 7 Flex cyclers, complemented with UgenTec FastFinder analysis and workflow software. Prior to extraction, samples in a non-virus inactivating medium, are first inactivated by the use of Sigma molecular medium (MM), in the context of biosafety measures. By the use of these Sigma MM tubes with a capture cap, the swab can be removed and placed directly on the pipetting robot. At the start of the extraction, the internal control, the MS2 bacteriophage, is added to the procedure. The assay targets three specific SARS-CoV-2 genes: the N-gene, ORF1ab and S, each with its own fluorescence group and result. The test result is reported as log copies/ml based on the quantification cycle (Cq) value of the N-gene, after the setup of a standard curve. Detection of two or more gene targets was considered a positive result.

### Statistical analyses

Paired data were used to construct a 2 × 2 contingency table (template in Table 1). Sensitivity was defined as the proportion of patients with SARS-CoV-2 as determined by TaqPath that tested positive with Aptima. Specificity was defined as the proportion of patients not carrying SARS-CoV-2 as determined by TaqPath that tested negative with Aptima. The overall per cent agreement was calculated as the proportion of concordant results (positive on both assays + negative on both assays) over total test results. Ninety-five per cent exact confidence intervals (95% CI) were calculated for all proportions. The agreement beyond chance was determined by Cohen’s kappa value (as defined by Fleiss [13]). Cohen’s kappa can be interpreted at different levels of agreement where (1.00 ≥ K > 0.80): excellent; (0.8 0 ≥ K > 0.60): good; (0.60 ≥ _K > 0.40): moderate; (0.40 ≥ _K > 0.20): fair; (0.20 ≥ K > 0.00): poor. The level of statistical significance was set at 0.05. Statistical analyses were performed with STATA version 14 (College Station, TX, USA).

**Table 1:** Contingency table (2 × 2) for the comparison of test performance for Aptima and TaqPath

		TaqPath* results		
		Positive	Negative	Total
Aptima results	Positive	A	B	A + B
	Negative	C	D	C + D
	Total	A + C	B + D	A + B + C + D

Parameter	Formula
Sensitivity of Aptima	100 × A/(A + C)
Specificity of Aptima	100 × D/(B + D)
Overall agreement	100 × (A + D)/(A + B + C + D)

*Reference test

## Results

### Initial testing with TaqPath as a standard reference assay

From the 90 negative samples from the panel, 90/90 tested negative on TaqPath. Out of the 90 samples that were initially classified as SARS-CoV-2 positive, 88/90 tested positive while two samples yielded a negative TaqPath result. These two samples were then reanalyzed on different assays. One of the two samples yielded a positive result with a Cycle Number (CN) value of 38.86 (true positive) on the Alinity m. The other sample was tested consistently negative when retested with Alinity m and another platform GeneXpert and therefore considered as a true negative.

As for the limit of detection, 4/40 of diluted clinical samples tested negative on TaqPath. The samples that were negative were from the 1:20 dilution (2 samples) and 1:50 dilution (2 samples). These four samples were retested with Alinity m and yielded positive results with CN values above 32 (Table 2).

**Table 2:** Tabulation of results of Aptima and the TaqPath on diluted clinical samples

Control dilution	TaqPath N-gene (Cq value)	Aptima results	Alinity m (CN value)
*Undiluted (1)*			
1:2	25.59	Positive	
1:10	28.70	Positive	
1:20	29.93	Positive	
1:50	29.26	Positive	
*Undiluted (2)*			
1:2	25.64	Positive	
1:10	28.93	Positive	
1:20	30.44	Positive	
1:50	31.08	Positive	
*Undiluted (3)*			
1:2	25.52	Positive	
1:10	28.82	Positive	
1:20	29.11	Positive	
1:50	30.66	Positive	
*Undiluted (4)*			
1:2	23.33	Positive	
1:10	26.53	Positive	
1:20	27.66	Positive	
1:50	28.89	Positive	
*Undiluted (5)*			
1:2	28.20	Positive	
1:10	31.41	Positive	
1:20	ND*	Positive	32.81
1:50	ND*	Positive	34.07
*Undiluted (6)*			
1:2	24.36	Positive	
1:10	27.41	Positive	
1:20	27.57	Positive	
1:50	28.67	Positive	
*Undiluted (7)*			
1:2	28.32	Positive	
1:10	32.00	Positive	
1:20	ND*	Positive	32.59
1:50	ND*	Positive	34.41
*Undiluted (8)*			
1:2	24.20	Positive	
1:10	26.91	Positive	
1:20	28.27	Positive	
1:50	29.47	Positive	
*Undiluted (9)*			
1:2	26.04	Positive	
1:10	29.13	Positive	
1:20	29.43	Positive	
1:50	30.27	Positive	
*Undiluted (10)*			
1:2	25.25	Positive	
1:10	28.06	Positive	
1:20	29.09	Positive	
1:50	30.11	Positive	

*ND* Not detected, *Cq* Quantitative cycle, *CN* Cycle Number, *N* nucleocapsid protein of SARS-CoV-2

*Samples that were not detected on TaqPath were retested with Alinity m

### Clinical sensitivity and specificity of Aptima compared to TaqPath

From the 88 samples that were positive for SARS-CoV-2 on TaqPath, 88/88 tested positive with Aptima corresponding to a sensitivity of 100.0% [95% CI 95.9–100.0]. 89/92 samples tested negative on Aptima yielding a specificity of 96.7% [95% CI 90.8–99.3]. The Cohen’s coefficient was κ = 0.967 [95% CI 0.929–1.000] with an overall percent agreement of 98.3% (Table 3). For the limit of detection, Aptima was able to detect all of the 40 diluted clinical samples with Cq values ≥ 32 (Table 2).

**Table 3:** Comparison of test performance of Aptima and the TaqPath using non-diluted clinical samples

		TaqPath		
		Positive	Negative	Total
Aptima SARS- CoV-2	Positive	88	3*	91
	Negative	0	89	81
	Total	88	92	180
Sensitivity		100.0%	(95% CI 95.9–100.0)	
Specificity		96.7%	(95% CI 90.8–99.3)	
Overall agreement		98.3%	(95% CI 95.2–99.7)	
Cohen’s Kappa (k) (95% CI)		0.967	(95% CI 0.929–1.000)	

*Only two of three Aptima positive/TaqPath negative samples were confirmed discordant

Aptima yielded positive results on three samples that were negative on TaqPath. One of the samples was the sample that was retested post hoc on Alinity M which yielded a positive result. The sample was missed by TaqPath due to the low viral load (close to the limit of detection for TaqPath) but was detected by Aptima. When accounting for this sample assuming that TaqPath missed a positive sample with low a viral load, the specificity of Aptima increased to 97.8% [95% CI 92.3–99.7].

## Discussion

In this study, we were able to demonstrate the test performance of the automated Aptima™ SARS-CoV-2 assay using clinical samples according to the VALCOR protocol. The analytical and clinical performance of the Hologic Aptima SARS-CoV-2 assay was evaluated against a panel of well-characterized samples and dilutions. Hologic Aptima SARS-CoV-2 assay showed very good concordance to the comparator, even in stress test conditions where samples were diluted 1:50 generating a larger than 6 Cq value shift of the comparator.

With the re-emergence of SARS-CoV-2 outbreaks across the globe in the summer of 2022 and the prospect of continued and increased epidemic activity, diagnostics and testing for SARS-CoV-2 will remain a cornerstone strategy in the control and surveillance of the COVID-19 outbreaks. It is, therefore, that assays available on the market must be thoroughly validated for high accuracy in screening and diagnosis of COVID-19.

When compared to the chosen reference standard (Taqpath), Aptima had a sensitivity of 100.0% [95% CI 95.9–100.0] and specificity of 96.7% [95% CI 90.8–99.3]. The overall percent agreement was 98.3% with an excellent Cohen’s coefficient of κ = 0.967 [95% CI 0.929–1.000]. Additionally, Aptima detected 40/40 of the diluted clinical samples corresponding to high analytical sensitivity (low limit of detection). Despite RT-PCR being the gold standard, previous studies have shown similar concordance and diagnostic performance with TMA assays relative to RT-PCR. In our study, Aptima was able to detect one true positive that was missed by the TaqPath corresponding to a comparable relative sensitivity of APTIMA versus TaqPath (89/88 = 1.01).

The Aptima assay offers several advantages as a testing method for SARS-CoV-2. These include the ease of use with a high level of automation and quick turnaround time with a nominal turnaround of 275 samples per 8 hours [14] (as per manufacturers specifications) and excellent test performance as demonstrated in this VALCOR study. Despite RT-PCR being widely used and considered the gold standard, other amplification methods such as TMA reduce the burden of labour-intensive manipulations that increase the turnover time of RT-PCR without compromising on the analytical sensitivity and specificity. A previous comparative study by Mostafa et al. [14] also reported excellent analytical performance of the Aptima assay, though on a significantly lower number of test-panel samples. Yet, any direct comparisons are hampered by the lack of international consensus on how to characterize SARS-CoV-2 assay performance.

Limitations of the study include the lack of detailed clinical information on patient samples, which may complete the clinical accuracy [15]. As the panel was collated during the first peak of the epidemic, there was a shortage of manpower and resources available to collect more complete information on the clinical status of patients. Additionally, the panel was collated before the emergence of new variants, it would have been beneficial to demonstrate the test performance of Aptima to detect newer variants of the SARS-CoV-2.

## Conclusion

In conclusion, we validated the analytical performance of the Aptima assay by using clinical samples collated through the VALCOR protocol. Aptima showed comparable performance to the TaqPath and was able to show a superior limit of detection.

## Data Availability

Datasets generated by VALCOR are stored locally and securely at Sciensano. Anonymized data can be made available by request to the corresponding author on a case-by-case basis pending approval from the information security coordinator at Sciensano.

## Abbreviations

SARS-CoV-2: Severe acute respiratory syndrome coronavirus 2
WHO: World Health Organization
COVID-19: Coronavirus disease 2019
NAATs: Nucleic acid amplification tests
RT-PCR: Reverse transcriptase-polymerase chain reaction
TMA: Transcription mediated amplification
EUA: Emergency use authorization
FDA: Food and drug administration
RNA: Ribonucleic acid
VALCOR: Validation of severe acute respiratory coronavirus 2 assays
VALGENT: Validation of HPV genotyping tests
UTM: Universal transport medium
PBS: Phosphate buffered saline
EC: Ethics Committee
LOD: Limit of detection
Cq: Quantification cycle
CN: Cycle number
